# Crystal structure of the 1:2 co-crystal of 1,3,6,8-tetra­aza­tri­cyclo­[4.3.1.1^3,8^]undecane (TATU) and 4-chloro­phenol (1/2)

**DOI:** 10.1107/S2056989016016546

**Published:** 2016-10-25

**Authors:** Augusto Rivera, Jicli José Rojas, Héctor Jairo Osorio, Jaime Ríos-Motta, Michael Bolte

**Affiliations:** a Universidad Nacional de Colombia, Sede Bogotá, Facultad de Ciencias, Departamento de Química, Cra 30 No. 45-03, Bogotá, Código Postal 110911, Colombia; b Universidad Nacional de Colombia, Sede Manizales, Manizales, Colombia; c Institut für Anorganische Chemie, J. W. Goethe-Universität Frankfurt, Max-von Laue-Str. 7, 60438 Frankfurt/Main, Germany

**Keywords:** crystal structure, co-crystalline adducts, hydrogen bonding, TATU

## Abstract

In the title compound, C_7_H_14_N_4_·2C_6_H_5_ClO, which crystallized with two crystallographically independent 4-chloro­phenol mol­ecules and one 1,3,6,8-tetra­aza­tri­cyclo­[4.3.1.1^3,8^]undecane (TATU) mol­ecule in the asymmetric unit, the independent components are linked by two O—H⋯N hydrogen bonds. The hydrogen-bond acceptor sites are two non-equivalent N atoms from the aminal cage structure, and the tricyclic system distorts by changing the C—N bond lengths. In the crystal, these hydrogen-bonded aggregates are linked into chains along the *c* axis by C—H⋯N hydrogen bonds. The crystal structure also features C—H⋯π contacts.

## Abstract 2

The components of the ternary co-crystalline adduct are linked by inter­molecular O–H⋯N hydrogen bonds.

## Chemical context   

Following our previous work on phenol–amine adducts based on cyclic aminal cages with phenol derivatives (Rivera *et al.*, 2015*a* ▸,*b* ▸,*c* ▸), we report herein the synthesis and crystal structure of the title 1:2 complex assembled through hydrogen-bonding inter­actions between the aminal cage, 1,3,6,8-tetra­aza­tri­cyclo [4.3.1.1^3,8^]undecane (TATU), with 4-chloro­phenol under solvent-free conditions at low temperature.
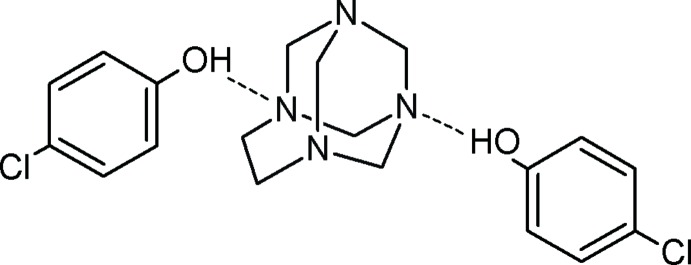


TATU, a small tricyclic aminal cage, is an inter­esting option for studying hydrogen-bonding situations as it has four nitro­gen atoms as potential hydrogen-bond acceptors. These N atoms have two different environments, N1 and N2 from the ethyl­ene fragment (NCH_2_CH_2_N) and N3 and N4 from the 1,1-*gem*-diaminic units. These present two discrete options for hydrogen bonding to the aminal cage. With different types of phenols, the preference for a particular hydrogen-bond-inter­action site depends strongly upon the lone-pair orbital hybridization of the nitro­gen atom (Rivera *et al.*, 2007 ▸). Studies on phenol complexes with tertiary amines in the solid state show that the proton transfer depends not only on the ΔpKa (pKa amine − pKa acid) value but also on steric and packing effects (Majerz & Sawka-Dobrowolska, 1996 ▸). In the structure found for the three-component aggregates observed here, both types of nitro­gen atom mentioned above are involved in hydrogen bonding with N1 and N3 acting as hydrogen-bond acceptors. The reaction to produce the co-crystal occurs efficiently in the solid state by grinding a mixture of finely powdered TATU and 4-chloro­phenol at room temperature; there are no by-products, and the work-up procedure is easy.

## Structural commentary   

**Figure 1:**
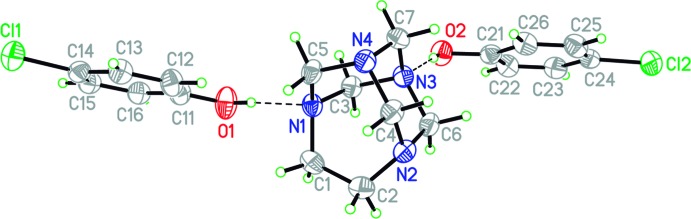
The mol­ecular structure of the title compound. Displacement ellipsoids are drawn at the 50% probability level. H atoms bonded to C atoms are omitted for clarity. Hydrogen bonds are drawn as dashed lines.

The mol­ecular structure of the title compound is illustrated in Fig. 1 ▸. The asymmetric unit comprises two crystallographically independent 4-chloro­phenol mol­ecules and one 1,3,6,8-tetra­aza­tri­cyclo­[4.3.1.1^3,8^]undecane (TATU) mol­ecule. The phenols are linked to the aminal cage by two O—H⋯N hydrogen bonds (Table 1 ▸), forming 2:1 hydrogen-bonded aggregates. This is similar to the situation observed in the structure of the 2:1 co-crystal of 4-nitro­phenol and TATU (Rivera *et al.*, 2015*a* ▸) which also crystallizes in the *P*2_1_/*c* space group and has two different types of N atom acting as the hydrogen-bond acceptors. The measured dimensions of the aminal cage structure in the present adduct are similar to the corresponding values in this related structure. The observed N—CH_2_ bond lengths are longer than those found in a co-crystal formed between TATU and hydo­quinone (Rivera *et al.*, 2007 ▸). This is presumably related to the formation of strong hydrogen bonds by the N1 and N3 hydrogen atoms.

A comparison of the O—H⋯N hydrogen bonds in the title compound with those found for the nitro­phenyl analogue (Rivera, *et al.*, 2015*a* ▸) reveal that both N⋯O distances are significantly longer in the current structure, suggesting that the hydrogen bonds may be somewhat weaker.

## Supra­molecular features   

**Figure 2:**
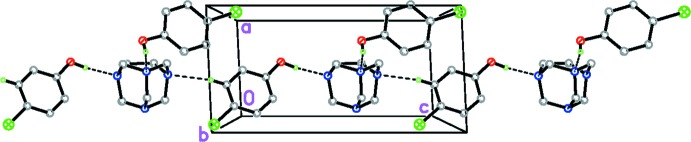
The crystal packing of the title compound, showing the chain that extends along the *c-*axis direction. C—H⋯N and O—H⋯N hydrogen bonds are drawn as dashed lines

In the crystal of title compound, O1—H1⋯N1 and C15—H15⋯N2 hydrogen bonds form columns of TATU mol­ecules and O1 chloro­phenol mol­ecules along the *c* axis, Fig. 2 ▸. The columns are linked by O2—H2⋯N3 hydrogen bonds on one side and C2—H2*A*⋯*Cg*8 contacts on the other (*Cg*8 is the centroid of the C11–C16 ring).

## Database survey   

Only three comparable structures were found in the Cambridge Structural Database (Groom *et al.*, 2016 ▸),, namely 1,3,6,8-tetra-aza­tri­cyclo­(4.3.1.1^3,8^)undecane hydro­quinone (HICTOD; Rivera *et al.*, 2007 ▸), 3,6,8-tri­aza-1-azoniatri­cyclo­[4.3.1.1^3,8^]undecane penta­chloro­phenolate monohydrate (OMODEA; Rivera *et al.*, 2011 ▸) and 4-nitro­phenol 1,3,6,8-tetra-aza­tri­cyclo­[4.3.1.1^3,8^]undecane (VUXMEI; Rivera *et al.*, 2015*a* ▸). These structures have already been discussed above.

## Synthesis and crystallization   

A mixture of 1,3,6,8-tetra­aza­tri­cyclo­[4.3.1.1^3,8^]undecane (TATU) (154 mg, 1 mmol) and 4-chloro­phenol (257 mg, 2 mmol) was mixed thoroughly in a mortar and then ground at room temperature for 15 min. Progress of the reaction was monitored by TLC. Crystals suitable for X-ray diffraction were obtained from a methanol solution upon slow evaporation of the solvent at room temperature (72% yield, m.p. = 334–336 K)

## Refinement   

Crystal data, data collection and structure refinement details are summarized in Table 2 ▸. All H atoms were located in difference electron-density maps. The hydroxyl H atoms were refined freely, while C-bound H atoms were fixed geometrically (C—H = 0.95, 0.98 or 0.99 Å) and refined using a riding model, with *U*_iso_(H) values set at 1.2*U*_eq_ of the parent atom (1.5 for methyl groups).

## Supplementary Material

Crystal structure: contains datablock(s) I. DOI: 10.1107/S2056989016016546/sj5511sup1.cif

Structure factors: contains datablock(s) I. DOI: 10.1107/S2056989016016546/sj5511Isup2.hkl

CCDC reference: 1510135

Additional supporting information: crystallographic information; 3D view; checkCIF report

## Figures and Tables

**Table 1:** Hydrogen-bond geometry (Å, °) *Cg*8 is the centroid of the C11–C16 ring.

*D*—H⋯*A*	*D*—H	H⋯*A*	*D*⋯*A*	*D*—H⋯*A*
O1—H1⋯N1	0.88 (3)	1.91 (3)	2.7824 (16)	172 (2)
O2—H2⋯N3	0.87 (2)	1.86 (2)	2.7186 (16)	167 (2)
C15—H15⋯N2^i^	0.95	2.56	3.4491 (18)	156
C2—H2*A*⋯*Cg*8^ii^	0.99	2.79	3.7348 (18)	160

Symmetry codes: (i) 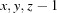; (ii) 
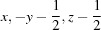
.

**Table 2:** Experimental details

Crystal data	
Chemical formula	C_7_H_14_N_4_·2C_6_H_5_ClO
*M*_r_	411.32
Crystal system, space group	Monoclinic, *P*2_1_/*c*
Temperature (K)	173
*a*, *b*, *c* (Å)	5.9495 (3), 27.6927 (8), 11.9402 (5)
β (°)	92.585 (3)
*V* (Å^3^)	1965.24 (14)
*Z*	4
Radiation type	Mo *K*α
μ (mm^−1^)	0.35
Crystal size (mm)	0.26 × 0.25 × 0.24

Data collection	
Diffractometer	STOE IPDS II two-circle
Absorption correction	Multi-scan (*X-AREA*; Stoe & Cie, 2001 ▸)
*T*_min_, *T*_max_	0.604, 1.000
No. of measured, independent and observed [*I* > 2σ(*I*)] reflections	37763, 4251, 4105
*R*_int_	0.045
(sin θ/λ)_max_ (Å^−1^)	0.640

Refinement	
*R*[*F* ^2^ > 2σ(*F* ^2^)], *wR*(*F* ^2^), *S*	0.040, 0.109, 1.09
No. of reflections	4251
No. of parameters	253
H-atom treatment	H atoms treated by a mixture of independent and constrained refinement
Δρ_max_, Δρ_min_ (e Å^−3^)	0.40, −0.26

Computer programs: *X-AREA* (Stoe & Cie, 2001 ▸), *SHELXS* (Sheldrick, 2008 ▸), *SHELXL2014/7* (Sheldrick, 2015 ▸) and *XP* in *SHELXTL-Plus* (Sheldrick, 2008 ▸).

## supplementary crystallographic information

### Crystal data

**Table:**

C_7_H_14_N_4_·2C_6_H_5_ClO	*F*(000) = 864
*M**_r_* = 411.32	*D*_x_ = 1.390 Mg m^−^^3^
Monoclinic, *P*2_1_/*c*	Mo *K*α radiation, λ = 0.71073 Å
*a* = 5.9495 (3) Å	Cell parameters from 37763 reflections
*b* = 27.6927 (8) Å	θ = 1.7–27.6°
*c* = 11.9402 (5) Å	µ = 0.35 mm^−^^1^
β = 92.585 (3)°	*T* = 173 K
*V* = 1965.24 (14) Å^3^	Block, colourless
*Z* = 4	0.26 × 0.25 × 0.24 mm

### Data collection

**Table:**

STOE IPDS II two-circle diffractometer	4105 reflections with *I* > 2σ(*I*)
Radiation source: Genix 3D IµS microfocus X-ray source	*R*_int_ = 0.045
ω scans	θ_max_ = 27.1°, θ_min_ = 1.9°
Absorption correction: multi-scan (X-Area; Stoe & Cie, 2001)	*h* = −7→7
*T*_min_ = 0.604, *T*_max_ = 1.000	*k* = −35→35
37763 measured reflections	*l* = −15→15
4251 independent reflections	

### Refinement

**Table:**

Refinement on *F*^2^	Hydrogen site location: mixed
Least-squares matrix: full	H atoms treated by a mixture of independent and constrained refinement
*R*[*F*^2^ > 2σ(*F*^2^)] = 0.040	*w* = 1/[σ^2^(*F*_o_^2^) + (0.0575*P*)^2^ + 0.6451*P*] where *P* = (*F*_o_^2^ + 2*F*_c_^2^)/3
*wR*(*F*^2^) = 0.109	(Δ/σ)_max_ = 0.002
*S* = 1.09	Δρ_max_ = 0.40 e Å^−^^3^
4251 reflections	Δρ_min_ = −0.26 e Å^−^^3^
253 parameters	Extinction correction: SHELXL-2014/7 (Sheldrick 2015), Fc^*^=kFc[1+0.001xFc^2^λ^3^/sin(2θ)]^-1/4^
0 restraints	Extinction coefficient: 0.062 (7)

### Special details

**Table:**

Geometry. All esds (except the esd in the dihedral angle between two l.s. planes) are estimated using the full covariance matrix. The cell esds are taken into account individually in the estimation of esds in distances, angles and torsion angles; correlations between esds in cell parameters are only used when they are defined by crystal symmetry. An approximate (isotropic) treatment of cell esds is used for estimating esds involving l.s. planes.

### Fractional atomic coordinates and isotropic or equivalent isotropic displacement parameters (Å^2^)

**Table:**

	*x*	*y*	*z*	*U*_iso_*/*U*_eq_	
Cl1	−0.04590 (7)	0.26962 (2)	−0.10224 (3)	0.04449 (15)	
O1	0.5641 (2)	0.35658 (5)	0.24199 (10)	0.0479 (3)	
H1	0.510 (4)	0.3572 (9)	0.310 (2)	0.073 (7)*	
C11	0.4191 (3)	0.33578 (5)	0.16530 (12)	0.0356 (3)	
C12	0.2099 (3)	0.31750 (6)	0.19246 (12)	0.0387 (3)	
H12	0.1644	0.3193	0.2676	0.046*	
C13	0.0679 (2)	0.29680 (5)	0.11068 (12)	0.0367 (3)	
H13	−0.0747	0.2845	0.1293	0.044*	
C14	0.1366 (2)	0.29428 (5)	0.00163 (12)	0.0339 (3)	
C15	0.3445 (2)	0.31185 (5)	−0.02693 (12)	0.0348 (3)	
H15	0.3898	0.3097	−0.1020	0.042*	
C16	0.4855 (2)	0.33256 (5)	0.05517 (12)	0.0350 (3)	
H16	0.6283	0.3447	0.0362	0.042*	
Cl2	1.00806 (8)	0.58275 (2)	1.06573 (4)	0.05235 (16)	
O2	0.7563 (2)	0.49889 (4)	0.62512 (9)	0.0406 (3)	
H2	0.655 (4)	0.4764 (8)	0.6299 (19)	0.059 (6)*	
C21	0.8062 (2)	0.51843 (5)	0.72828 (12)	0.0328 (3)	
C22	1.0146 (2)	0.54078 (5)	0.74570 (13)	0.0376 (3)	
H22	1.1157	0.5421	0.6864	0.045*	
C23	1.0755 (2)	0.56101 (5)	0.84860 (14)	0.0391 (3)	
H23	1.2175	0.5763	0.8601	0.047*	
C24	0.9276 (3)	0.55872 (5)	0.93448 (13)	0.0367 (3)	
C25	0.7182 (3)	0.53749 (5)	0.91821 (14)	0.0406 (3)	
H25	0.6167	0.5367	0.9774	0.049*	
C26	0.6576 (2)	0.51743 (5)	0.81477 (14)	0.0382 (3)	
H26	0.5137	0.5029	0.8030	0.046*	
N1	0.43277 (18)	0.36094 (4)	0.46248 (10)	0.0308 (3)	
N2	0.44381 (19)	0.33973 (4)	0.69815 (10)	0.0324 (3)	
N3	0.47481 (19)	0.42152 (4)	0.61627 (10)	0.0310 (3)	
N4	0.12298 (18)	0.37751 (4)	0.59049 (9)	0.0310 (3)	
C1	0.5461 (3)	0.31658 (5)	0.49947 (13)	0.0385 (3)	
H1A	0.7030	0.3179	0.4754	0.046*	
H1B	0.4717	0.2892	0.4595	0.046*	
C2	0.5526 (3)	0.30537 (6)	0.62630 (14)	0.0393 (3)	
H2A	0.4821	0.2734	0.6366	0.047*	
H2B	0.7122	0.3026	0.6527	0.047*	
C3	0.5404 (2)	0.40561 (5)	0.50412 (11)	0.0307 (3)	
H3A	0.5042	0.4318	0.4498	0.037*	
H3B	0.7055	0.4009	0.5064	0.037*	
C4	0.1997 (2)	0.34370 (5)	0.67956 (11)	0.0330 (3)	
H4A	0.1345	0.3539	0.7506	0.040*	
H4B	0.1389	0.3113	0.6609	0.040*	
C5	0.1885 (2)	0.36212 (5)	0.47928 (11)	0.0329 (3)	
H5A	0.1172	0.3841	0.4228	0.039*	
H5B	0.1268	0.3294	0.4646	0.039*	
C6	0.5478 (2)	0.38714 (5)	0.70586 (11)	0.0338 (3)	
H6A	0.7129	0.3831	0.7043	0.041*	
H6B	0.5145	0.4015	0.7792	0.041*	
C7	0.2262 (2)	0.42473 (5)	0.61453 (12)	0.0339 (3)	
H7A	0.1786	0.4365	0.6881	0.041*	
H7B	0.1737	0.4482	0.5566	0.041*	

### Atomic displacement parameters (Å^2^)

**Table:**

	*U*^11^	*U*^22^	*U*^33^	*U*^12^	*U*^13^	*U*^23^
Cl1	0.0475 (2)	0.0507 (2)	0.0351 (2)	−0.00710 (16)	0.00008 (15)	−0.00103 (15)
O1	0.0452 (6)	0.0666 (8)	0.0328 (6)	−0.0153 (5)	0.0091 (5)	−0.0071 (5)
C11	0.0382 (7)	0.0384 (7)	0.0307 (7)	−0.0009 (6)	0.0052 (5)	−0.0002 (5)
C12	0.0437 (8)	0.0445 (8)	0.0288 (7)	−0.0034 (6)	0.0106 (6)	−0.0010 (6)
C13	0.0364 (7)	0.0390 (7)	0.0353 (7)	−0.0010 (6)	0.0087 (6)	0.0023 (6)
C14	0.0385 (7)	0.0332 (7)	0.0301 (7)	0.0036 (5)	0.0016 (5)	0.0031 (5)
C15	0.0396 (7)	0.0375 (7)	0.0278 (6)	0.0049 (6)	0.0070 (5)	0.0053 (5)
C16	0.0365 (7)	0.0377 (7)	0.0314 (7)	0.0014 (6)	0.0084 (5)	0.0040 (5)
Cl2	0.0697 (3)	0.0430 (2)	0.0434 (2)	0.00721 (18)	−0.00754 (19)	−0.00895 (16)
O2	0.0466 (6)	0.0377 (5)	0.0373 (6)	−0.0086 (5)	0.0001 (4)	0.0005 (4)
C21	0.0355 (7)	0.0269 (6)	0.0360 (7)	0.0002 (5)	0.0003 (5)	0.0035 (5)
C22	0.0357 (7)	0.0348 (7)	0.0428 (8)	−0.0045 (6)	0.0079 (6)	0.0012 (6)
C23	0.0342 (7)	0.0344 (7)	0.0486 (8)	−0.0033 (5)	−0.0004 (6)	−0.0018 (6)
C24	0.0437 (8)	0.0279 (6)	0.0381 (7)	0.0051 (5)	−0.0018 (6)	−0.0006 (5)
C25	0.0431 (8)	0.0358 (7)	0.0437 (8)	−0.0002 (6)	0.0104 (6)	0.0003 (6)
C26	0.0317 (7)	0.0348 (7)	0.0485 (8)	−0.0032 (5)	0.0051 (6)	−0.0005 (6)
N1	0.0275 (5)	0.0360 (6)	0.0291 (5)	−0.0013 (4)	0.0036 (4)	−0.0042 (4)
N2	0.0306 (6)	0.0366 (6)	0.0300 (6)	0.0007 (4)	0.0005 (4)	0.0031 (5)
N3	0.0308 (6)	0.0320 (6)	0.0301 (6)	−0.0003 (4)	0.0017 (4)	−0.0026 (4)
N4	0.0257 (5)	0.0392 (6)	0.0282 (6)	0.0020 (4)	0.0030 (4)	−0.0008 (5)
C1	0.0364 (7)	0.0358 (7)	0.0438 (8)	0.0028 (6)	0.0076 (6)	−0.0058 (6)
C2	0.0353 (7)	0.0367 (7)	0.0459 (8)	0.0047 (6)	0.0016 (6)	0.0015 (6)
C3	0.0291 (6)	0.0340 (7)	0.0294 (6)	−0.0022 (5)	0.0041 (5)	0.0003 (5)
C4	0.0294 (6)	0.0400 (7)	0.0298 (6)	−0.0017 (5)	0.0051 (5)	0.0032 (5)
C5	0.0259 (6)	0.0457 (8)	0.0270 (6)	−0.0020 (5)	0.0005 (5)	−0.0027 (5)
C6	0.0328 (7)	0.0403 (7)	0.0280 (6)	−0.0016 (5)	−0.0030 (5)	−0.0024 (5)
C7	0.0318 (7)	0.0347 (7)	0.0354 (7)	0.0057 (5)	0.0038 (5)	−0.0025 (5)

### Geometric parameters (Å, º)

**Table:**

Cl1—C14	1.7497 (15)	N1—C3	1.4692 (17)
O1—C11	1.3579 (19)	N1—C5	1.4766 (17)
O1—H1	0.88 (3)	N2—C6	1.4524 (18)
C11—C16	1.3929 (19)	N2—C2	1.4529 (19)
C11—C12	1.395 (2)	N2—C4	1.4635 (17)
C12—C13	1.386 (2)	N3—C3	1.4789 (17)
C12—H12	0.9500	N3—C7	1.4808 (17)
C13—C14	1.384 (2)	N3—C6	1.4824 (18)
C13—H13	0.9500	N4—C5	1.4639 (17)
C14—C15	1.386 (2)	N4—C7	1.4675 (18)
C15—C16	1.386 (2)	N4—C4	1.4739 (17)
C15—H15	0.9500	C1—C2	1.545 (2)
C16—H16	0.9500	C1—H1A	0.9900
Cl2—C24	1.7494 (15)	C1—H1B	0.9900
O2—C21	1.3657 (18)	C2—H2A	0.9900
O2—H2	0.87 (2)	C2—H2B	0.9900
C21—C26	1.390 (2)	C3—H3A	0.9900
C21—C22	1.393 (2)	C3—H3B	0.9900
C22—C23	1.384 (2)	C4—H4A	0.9900
C22—H22	0.9500	C4—H4B	0.9900
C23—C24	1.382 (2)	C5—H5A	0.9900
C23—H23	0.9500	C5—H5B	0.9900
C24—C25	1.384 (2)	C6—H6A	0.9900
C25—C26	1.387 (2)	C6—H6B	0.9900
C25—H25	0.9500	C7—H7A	0.9900
C26—H26	0.9500	C7—H7B	0.9900
N1—C1	1.4601 (19)		
			
C11—O1—H1	112.3 (16)	C7—N3—C6	107.96 (11)
O1—C11—C16	117.72 (13)	C5—N4—C7	108.10 (11)
O1—C11—C12	122.92 (13)	C5—N4—C4	112.53 (11)
C16—C11—C12	119.36 (14)	C7—N4—C4	108.15 (11)
C13—C12—C11	120.54 (13)	N1—C1—C2	117.17 (12)
C13—C12—H12	119.7	N1—C1—H1A	108.0
C11—C12—H12	119.7	C2—C1—H1A	108.0
C14—C13—C12	119.15 (13)	N1—C1—H1B	108.0
C14—C13—H13	120.4	C2—C1—H1B	108.0
C12—C13—H13	120.4	H1A—C1—H1B	107.2
C13—C14—C15	121.22 (14)	N2—C2—C1	117.06 (12)
C13—C14—Cl1	119.11 (12)	N2—C2—H2A	108.0
C15—C14—Cl1	119.65 (11)	C1—C2—H2A	108.0
C16—C15—C14	119.34 (13)	N2—C2—H2B	108.0
C16—C15—H15	120.3	C1—C2—H2B	108.0
C14—C15—H15	120.3	H2A—C2—H2B	107.3
C15—C16—C11	120.39 (13)	N1—C3—N3	115.37 (11)
C15—C16—H16	119.8	N1—C3—H3A	108.4
C11—C16—H16	119.8	N3—C3—H3A	108.4
C21—O2—H2	110.4 (15)	N1—C3—H3B	108.4
O2—C21—C26	122.85 (13)	N3—C3—H3B	108.4
O2—C21—C22	117.81 (13)	H3A—C3—H3B	107.5
C26—C21—C22	119.33 (14)	N2—C4—N4	115.47 (11)
C23—C22—C21	120.55 (14)	N2—C4—H4A	108.4
C23—C22—H22	119.7	N4—C4—H4A	108.4
C21—C22—H22	119.7	N2—C4—H4B	108.4
C24—C23—C22	119.35 (14)	N4—C4—H4B	108.4
C24—C23—H23	120.3	H4A—C4—H4B	107.5
C22—C23—H23	120.3	N4—C5—N1	115.69 (11)
C23—C24—C25	120.97 (14)	N4—C5—H5A	108.4
C23—C24—Cl2	119.32 (12)	N1—C5—H5A	108.4
C25—C24—Cl2	119.71 (12)	N4—C5—H5B	108.4
C24—C25—C26	119.44 (14)	N1—C5—H5B	108.4
C24—C25—H25	120.3	H5A—C5—H5B	107.4
C26—C25—H25	120.3	N2—C6—N3	115.14 (11)
C25—C26—C21	120.33 (14)	N2—C6—H6A	108.5
C25—C26—H26	119.8	N3—C6—H6A	108.5
C21—C26—H26	119.8	N2—C6—H6B	108.5
C1—N1—C3	114.69 (11)	N3—C6—H6B	108.5
C1—N1—C5	114.92 (11)	H6A—C6—H6B	107.5
C3—N1—C5	110.62 (11)	N4—C7—N3	111.00 (11)
C6—N2—C2	115.45 (12)	N4—C7—H7A	109.4
C6—N2—C4	111.01 (11)	N3—C7—H7A	109.4
C2—N2—C4	115.17 (12)	N4—C7—H7B	109.4
C3—N3—C7	108.03 (10)	N3—C7—H7B	109.4
C3—N3—C6	112.41 (11)	H7A—C7—H7B	108.0
			
O1—C11—C12—C13	179.72 (15)	C4—N2—C2—C1	65.17 (17)
C16—C11—C12—C13	−0.7 (2)	N1—C1—C2—N2	0.5 (2)
C11—C12—C13—C14	0.3 (2)	C1—N1—C3—N3	−85.46 (14)
C12—C13—C14—C15	0.3 (2)	C5—N1—C3—N3	46.49 (15)
C12—C13—C14—Cl1	−178.29 (12)	C7—N3—C3—N1	−53.85 (15)
C13—C14—C15—C16	−0.4 (2)	C6—N3—C3—N1	65.16 (15)
Cl1—C14—C15—C16	178.18 (11)	C6—N2—C4—N4	47.85 (15)
C14—C15—C16—C11	−0.1 (2)	C2—N2—C4—N4	−85.71 (15)
O1—C11—C16—C15	−179.79 (14)	C5—N4—C4—N2	65.04 (15)
C12—C11—C16—C15	0.6 (2)	C7—N4—C4—N2	−54.30 (15)
O2—C21—C22—C23	−179.63 (13)	C7—N4—C5—N1	54.75 (15)
C26—C21—C22—C23	1.2 (2)	C4—N4—C5—N1	−64.62 (16)
C21—C22—C23—C24	0.2 (2)	C1—N1—C5—N4	84.81 (15)
C22—C23—C24—C25	−1.5 (2)	C3—N1—C5—N4	−47.03 (16)
C22—C23—C24—Cl2	178.15 (12)	C2—N2—C6—N3	85.50 (15)
C23—C24—C25—C26	1.2 (2)	C4—N2—C6—N3	−47.92 (15)
Cl2—C24—C25—C26	−178.40 (12)	C3—N3—C6—N2	−64.54 (15)
C24—C25—C26—C21	0.3 (2)	C7—N3—C6—N2	54.51 (15)
O2—C21—C26—C25	179.42 (14)	C5—N4—C7—N3	−61.56 (14)
C22—C21—C26—C25	−1.5 (2)	C4—N4—C7—N3	60.53 (14)
C3—N1—C1—C2	64.76 (16)	C3—N3—C7—N4	61.15 (14)
C5—N1—C1—C2	−65.11 (16)	C6—N3—C7—N4	−60.65 (14)
C6—N2—C2—C1	−66.31 (17)		

### Hydrogen-bond geometry (Å, º)

Cg8 is the centroid of the C11–C16 ring.

**Table:**

*D*—H···*A*	*D*—H	H···*A*	*D*···*A*	*D*—H···*A*
O1—H1···N1	0.88 (3)	1.91 (3)	2.7824 (16)	172 (2)
O2—H2···N3	0.87 (2)	1.86 (2)	2.7186 (16)	167 (2)
C15—H15···N2^i^	0.95	2.56	3.4491 (18)	156
C2—H2*A*···*Cg*8^ii^	0.99	2.79	3.7348 (18)	160

Symmetry codes: (i) *x*, *y*, *z*−1; (ii) *x*, −*y*−1/2, *z*−1/2.
